# Myoblast mitochondrial respiration is decreased in chronic binge alcohol administered simian immunodeficiency virus‐infected antiretroviral‐treated rhesus macaques

**DOI:** 10.14814/phy2.13625

**Published:** 2018-03-04

**Authors:** Anthony A. Duplanty, Robert W. Siggins, Timothy Allerton, Liz Simon, Patricia E. Molina

**Affiliations:** ^1^ Department of Physiology Comprehensive Alcohol Research Center, Alcohol and Drug Abuse Center of Excellence Louisiana State University Health Sciences Center New Orleans Louisiana

**Keywords:** Chronic alcohol, HIV, mitochondria, SIV

## Abstract

Work from our group demonstrated that chronic binge alcohol (CBA)‐induces mitochondrial gene dysregulation at end‐stage disease of simian immunodeficiency virus (SIV) infection in antiretroviral therapy (ART) naïve rhesus macaques. Alterations in gene expression can disrupt mitochondrial homeostasis and in turn contribute to the risk of metabolic comorbidities characterized by loss of skeletal muscle (SKM) functional mass that are associated with CBA, human immunodeficiency virus (HIV) infection, and prolonged ART. The aim of this study was to examine the interaction of CBA and ART on SKM fiber oxidative capacity and myoblast mitochondrial respiration in asymptomatic SIV‐infected macaques. SKM biopsies were obtained and myoblasts isolated at baseline and 11 months post‐SIV infection from CBA/SIV/ART+ and from sucrose (SUC)‐treated SIV‐infected (SUC/SIV/ART+) macaques. CBA and ART decreased succinate dehydrogenase (SDH) activity in type 1 and type 2b fibers as determined by immunohistochemistry. Myoblasts isolated from CBA/SIV/ART+ macaques showed decreased maximal oxygen consumption rate (OCR) compared to myoblasts from control macaques. Maximal OCR was significantly increased in control myoblasts following incubation with formoterol, a beta adrenergic agonist, and this was associated with increased PGC‐1α expression and mtDNA quantity. Additionally, formoterol treatment of myoblasts isolated from CBA/SIV/ART+ macaques partially restored maximal OCR to levels not significantly different from control. These results show that CBA in combination with ART impairs myoblast mitochondrial homeostasis in SIV‐infected macaques. Moreover, our findings suggest that adrenergic agonists can potentially ameliorate mitochondrial dysfunction. Future studies will elucidate whether physical exercise in HIV patients with alcohol use disorder can improve mitochondrial health.

## Introduction

Antiretroviral therapy (ART) has altered the course of HIV disease progression from an acute to a chronic illness and persons living with HIV (PLWH) have a longer lifespan (Deeks et al. 2013). Extended survival is associated with an increased incidence of age‐related comorbidities including skeletal muscle myopathy, metabolic syndrome, cardiovascular, and renal disease (Peters et al. 2013). In addition, PLWH have a higher prevalence of alcohol use disorder (AUD) and among 1.15 million PLWH in the US, more than 30% meet criteria for AUD (Gordon et al. 2006). Chronic alcohol consumption and HIV both result in significant skeletal muscle (SKM) derangements including atrophy, weakness, and dysfunction (Preedy and Peters 1994; Scott et al. 2007; Vary and Lang 2008; Clary et al. 2011; Richert et al. 2011). Mitochondria form a dynamic network in the SKM and are central to cellular metabolism, control production of ATP, is a source for physiological reactive oxygen species (ROS) and maintain calcium homeostasis, and mitochondrial dysfunction leads to loss of SKM functional capacity (Russell et al. 2014; Johnson et al. 2013).

Our laboratory has previously demonstrated that chronic binge alcohol (CBA) exacerbates skeletal muscle wasting at end‐stage simian acquired immune deficiency syndrome (SAIDS) in untreated macaques through mechanisms that include increased proteasomal activity, a profibrotic milieu, decreased antioxidant capacity and dysregulation of genetic and epigenetic regulatory networks of energy homeostasis (Molina et al. 2006, 2008; LeCapitaine et al. 2011; Dodd et al. 2014; Simon et al. 2015). We also recently reported that CBA impairs expression of genes associated with mitochondrial biogenesis and mitophagy (Duplanty et al. 2017).

Chronic alcohol consumption, HIV, and ART have all been implicated in impaired mitochondrial bioenergetics (Carbonari et al. 1997; Koczor and Lewis 2010; Selvaraj et al. 2014; Tahrir et al. 2018) and this affects tissues that heavily rely on oxidative phosphorylation for energy. Moreover, HIV disease accelerates aging (Onen and Overton 2011; Pathai et al. 2014; Katz et al. 2015), and one of the major age‐related changes is a decrease in whole‐body oxidative capacity related to decrease in muscle mass (Peterson et al. 2012) and cardiac output (Russ and Kent‐Braun 2004). SKM oxidative capacity depends on the number and function of mitochondria (Short and Nair 2001). Additionally, dysregulated mitochondrial respiration and biogenesis is associated with increased ROS generation and sarcopenia (Johnson et al. 2013; Joseph et al. 2013). Markers of SKM mitochondrial function including enzyme expression and activity of succinate dehydrogenase (SDH), mitochondrial respiration rates, mitochondrial biogenesis and abundance, genes, and proteins associated with mitochondrial function (Short and Nair 2001) have been shown to decrease with aging and disease states. There is a growing body of evidence suggesting that exercise interventions, especially aerobic exercise, enhances SKM function by mitochondrial adaptations, including enhanced biogenesis (Hood 2009; Hood et al. 2011; Konopka et al. 2014; Robinson et al. 2017). In vitro incubation with formoterol, a long‐acting β2‐adrenergic receptor agonist, has been shown to stimulate expression of PPARγ coactivator‐1 family of genes (PGC‐1α) and increase mitochondrial respiration and biogenesis, and has been used as an “exercise mimetic” to examine mitochondrial stimulated biological responses (Miura et al. 2007; Wills et al. 2012).

This study aimed to further characterize the mechanisms of SIV‐, CBA‐ and ART‐mediated loss of SKM functional mass in rhesus macaques. Our results demonstrate the potential role of CBA to decrease mitochondrial respiratory function and oxidative capacity, which can be at least partially restored by formoterol. These findings strongly support implementation of behavioral or therapeutic interventions to potentially improve SKM functional mass in PLWH with AUD.

## Methods

All animal experiments used in this study were approved by the Institutional Animal Care and Use Committee at both Tulane National Primate Research Center (TNPRC) in Covington and Louisiana State University Health Sciences Center (LSUHSC) in New Orleans, Louisiana, and adhered to the National Institutes of Health guidelines for the care and use of experimental animals. Four‐ to six‐year‐old male rhesus macaques *(Macaca mulatta)* were used for the study. The detailed experimental design, virological, immune, and metabolic data collected from the parent longitudinal study have been previously published (Molina et al. 2014; Ford et al. 2016; Simon et al. 2017b,c).

Briefly, macaques were administered daily intragastric alcohol (13–14 g of ethanol/kg body weight/week; 30% w/v water) or isocaloric sucrose for 3 months prior to SIV infection, and continued for the duration of the study (Bagby et al. 2006; Molina et al. 2014). This approach of intragastric delivery was selected to reduce experimental variability and ensure chronic binge‐like intoxicating blood alcohol concentrations of 50–60 mmol/L at about 2 h after starting the infusion. The calories provided by alcohol and sucrose averaged 15% of total caloric intake. Animals were provided monkey chow ad libitum (Lab Fiber plus Primate diet DT; PMI Nutrition International, St. Louis, MO) and supplemented with fruits, vitamins, and Noyes treats (Research Diets, New Brunswick, NJ). Following 3 months of daily alcohol or sucrose administration, animals were SIV‐inoculated. After 2.5 months of SIV infection, animals were randomized to receive ART (provided by Gilead Sciences, Inc.) consisting of daily subcutaneous injections of 20 mg/kg of Tenofovir (TFV, 9‐[R‐2‐(phosphonomethoxy) propyl] adenine, PMPA) and 30 mg/kg of Emtricitabine (FTC) (Molina et al. 2014; Ford et al. 2016; Simon et al. 2017b) for a total of four treatment groups: SUC/SIV/ART‐ (*n* = 4); SUC/SIV/ART+ (*n* = 7); CBA/SIV/ART‐ (*n* = 6); and CBA/SIV/ART+ (*n* = 7). Skeletal muscle (SKM) samples were obtained by biopsy at baseline before the start of the study (CTRL, *n* = 6) and excised at necropsy (11.5 months post‐SIV). SKM was used for myoblast isolation from fresh tissue (Simon et al. 2014, 2017a), and snap frozen and stored at −80°C for biochemical analysis. Because we were unable to expand two lines from the four myoblasts lines in the SUC/SIV/ART‐ group, only SUC/SIV/ART+ (*n* = 4); and CBA/SIV/ART+ (*n* = 4) primary cell lines were used for these studies, precluding us from elucidating the overall effects of CBA and ART. Experiments were performed in triplicates for each primary cell line.

### Skeletal muscle succinate dehydrogenase activity

Serial cross sections (10 μm) from snap frozen quadriceps muscle tissue were stained for SDH activity as previously described (Sheehan 1987). Briefly, sections were incubated in staining medium containing sodium succinate, nitro blue tetrazolium (NBT) dissolved in phosphate buffer for 60 min at 37°C. They were washed in increasing concentrations of acetone to remove excess NBT, washed in water and mounted with an aqueous mounting medium. Bright‐field images for analysis of SDH activity were obtained at a 20× magnification in a blinded fashion of at least 10 fields per slide using a Nikon Eclipse TE2000‐U microscope (Nikon instruments Inc., Melville, NY) and NIS Elements Imaging Software (Version 3.22.11).

### Mitochondrial respiration

Primary myoblast isolation and confirmation of myoblast phenotype has been previously published (Simon et al. 2014, 2017b). Myoblasts were seeded (60,000 cells/well) in triplicates on a Seahorse culture plate and cultured in Ham's‐F12 media with 10% FBS, 2% l‐Glutamine and 2.5 ng/mL hFGF for 24 h. Cells from passage 3 to passage 4 were used for all experiments described. Media were then changed to XF Assay Medium [XF Base Media (Cat No. 102353‐100, Seahorse Bioscience) with sodium pyruvate, l‐glutamine and glucose) for 1 h before mitochondrial function was assessed by measuring cellular respiration rates with a Seahorse Flux Analyzer XF‐24 (Agilent technologies, Santa Clara, CA) as specified by the manufacturer. Respiratory parameters (basal and maximal oxygen consumption rates (OCR), proton leak, and spare respiratory capacity) were assessed in response to addition of oligomycin (1 μmol/L), carbonyl cyanide‐p‐trifluoromethoxyphenylhydrazone (FCCP; 2 μmol/L), and rotenone/antimycin A (0.5 μmol/L). Values were normalized to protein content for each well and represented as normalized values to the controls. Myoblasts isolated from control and CBA/SIV/ART+ macaques were concomitantly cultured with Formoterol (30 nmol/L) for 24 h.

### Mitochondrial DNA copy number

To determine the effects of formoterol on mitochondrial biogenesis, control and CBA/SIV/ART+ macaque myoblasts were seeded (60,000 cells/well) and cultured in media containing 30 nmol/L formoterol (Sigma‐Aldrich, St. Louis, MO) or media for 6 h. β2 receptors are expressed by myoblasts. At completion of incubation, RNA was extracted with RNeasy Mini Universal kit (Qiagen, Valencia, CA) for determining PGC‐1α expression. To determine formoterol effects on mtDNA copy number, control and CBA/SIV/ART+ macaque myoblasts were cultured in media containing 30 nmol/L formoterol or media for 24 h. DNA was extracted using DNeasy Blood & Tissue kit (Qiagen) and mtDNA copy number (mtDNA/Nuclear DNA: Dloop/B2M) (Jackson et al. 2012) determined. The primers are the following, Dloop Forward 5′‐CAA GAT CGC CCA CAC GTT C‐3′ and Reverse 5′‐ AAA TCT CCC GTG ACT GGT TA‐3′; B2M Forward 5′‐TGT AAG CAG CAT CAT GGA GGT ‐3′ and Reverse 5′‐TGT TCT CCA CAT AGT GAG GGT‐3′.

### Statistical analyses

Data are displayed as mean ± SEM. Statistical analysis of SDH activity was determined using two‐way ANOVA followed by Bonferroni post hoc test. Statistical analysis of Seahorse OCR was determined using one‐way ANOVA followed by Tukey's posttest or unpaired *t*‐tests. Statistical analyses were performed using Graph Pad Prism 5 (Graph Pad Software, Inc.; La Jolla, CA). Statistical significance was set at *P* ≤ 0.05.

## Results

### Succinate dehydrogenase activity in skeletal muscle

**Figure 1:**
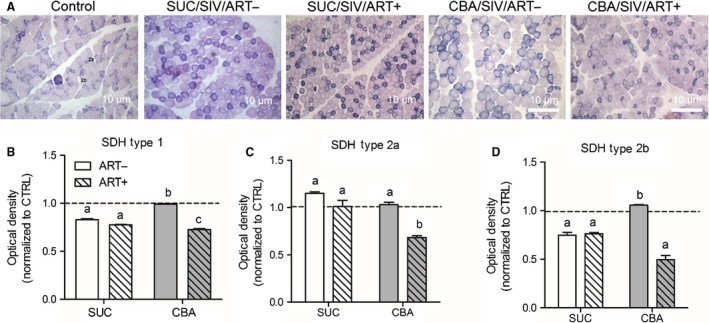
Succinate dehydrogenase activity. Skeletal muscle (SKM) tissue isolated from sucrose (SUC)‐ or chronic binge alcohol (CBA)‐administered macaques with and without antiretroviral therapy (ART) was stained for succinate dehydrogenase (SDH) activity and compared to naïve control (CTRL) macaque SKM tissue. (A) Representative micrographs of SDH activity in the muscle. (B) There was a significant CBA × ART interaction (*P* < 0.05) to decrease SDH activity in Type 1 and Type 2b skeletal muscle fiber types. (C) There was a main effect of CBA to decrease SDH activity in type 2a fibers. (D) There was a main effect of ART to decrease SDH activity in type 2b fibers. Optical density quantification was normalized to control (Dotted line; *n* = 4) for each of the following groups: SUC/SIV/ART‐ (white open bars, *n* = 5), SUC/SIV/ART+ (white hatched bars, *n* = 3), CBA/SIV/ART‐ (gray bars, *n* = 3), and CBA/SIV/ART+ (gray hatched bars, *n* = 4). Data are expressed as mean ± SEM.

SDH activity in type 1 fibers and Type 2b (fast twitch glycolytic) fibers in the SKM of SUC/SIV/ART‐, SUC/SIV/ART+, and CBA/SIV/ART+ was significantly lower than that of controls (Fig. 1B and C). SDH activity in Type 2a (fast twitch oxidative) fibers of CBA/SIV/ART+ animals was significantly lower than that of controls, SUC/SIV/ART‐, SUC/SIV/ART+, and CBA/SIV/ART+ animals (Fig. 1C). Overall, the mean decrease in SDH activity in all fibers was greatest in the CBA/SIV/ART+ group.

### Myoblast maximal oxygen consumption rate

**Figure 2:**
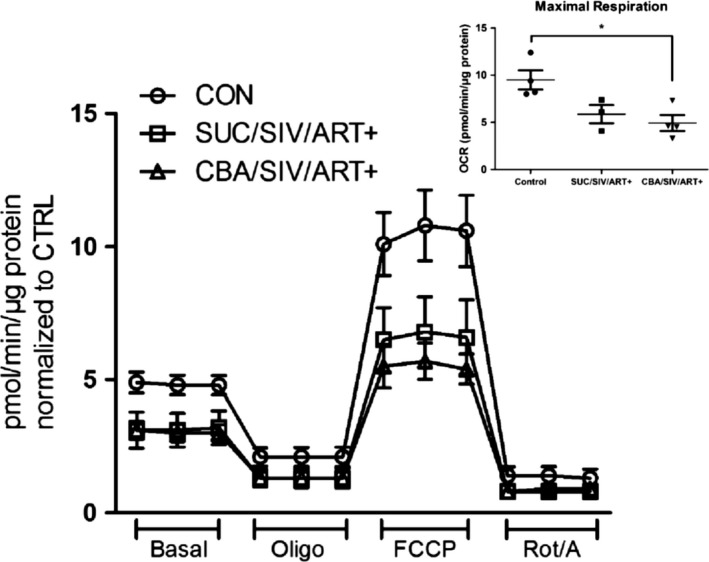
Myoblast oxygen consumption rates (OCR). OCR of macaque myoblasts were measured using the Seahorse XF24e extracellular flux analyzer in response to 1.0 μmol/L oligomycin (Oligo), 2.0 μmol/L carbonyl cyanide‐p‐trifluoromethoxyphenylhydrazone (FCCP), and 0.5 μmol/L Rotenone/antimycin A (Rot/A). There were no significant differences in Basal OCR. Maximal OCR was significantly lower in CBA/SIV/ART+ compared to CTRL (Inset). *P < 0.05. Data are expressed as mean ± SEM. *N* = 3–4/group.

Using the Seahorse XF24 analyzer multiple parameters of mitochondrial bioenergetics were determined in myoblasts. Basal oxygen consumption rate (OCR) was decreased in SUC/SIV/ART+ and CBA/SIV/ART+ myoblasts compared to naïve control myoblasts, but this difference failed to achieve statistical significance (Fig. 2). Maximal OCR of myoblasts isolated from SUC/SIV/ART+ and CBA/SIV/ART+ macaques were lower than that of myoblasts isolated from controls, but reached statistical significance only between CBA/SIV/ART+ and controls (Fig. 2 and inset). There were no statistically significant differences in other respiratory measures (Table 1).

**Table 1:** Oxygen consumption rates (OCR) of myoblasts measured using the XF24 extracellular flux analyzer. Results are normalized to control myoblasts

Respiratory parameters	Control	SUC/SIV/ART+	CBA/SIV/ART+
Basal respiration	1 ± 0.07	0.96 ± 0.34	0.59 ± 0.14
Proton leak	1 ± 0.18	1.58 ± 0.84	0.59 ± 0.12
Maximal respiration	1 ± 0.14	0.71 ± 0.16	0.5 ± 0.08
Spare respiratory capacity	1 ± 0.26	0.57 ± 0.10	0.44 ± 0.08
Nonmitochondrial respiration	1 ± 0.27	−0.42 ± 0.94	0.54 ± 0.13
ATP Production	1 ± 0.10	0.8 ± 0.22	0.6 ± 0.13

### Formoterol stimulation of mitochondrial responses

**Figure 3:**
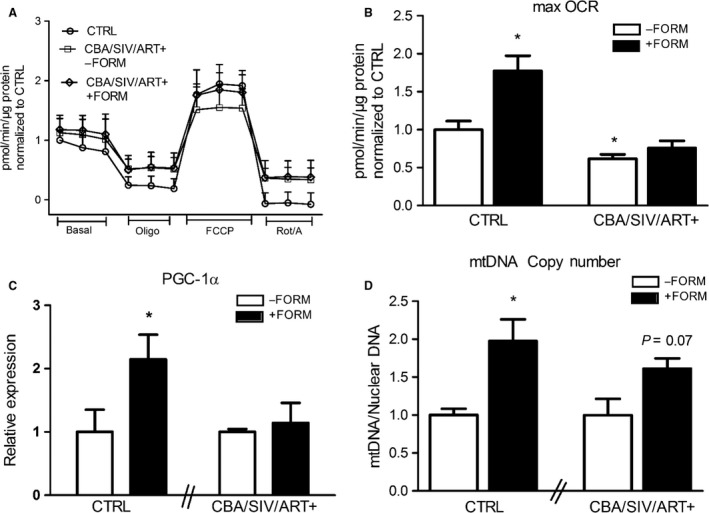
Control and CBA/SIV/ART+ myoblast formoterol treatment. (A) Oxygen consumption rates (OCR) of myoblasts measured using the Seahorse XF24e extracellular flux analyzer in response to 1.0 μmol/L oligomycin (Oligo), 2.0 μmol/L carbonyl cyanide‐p‐trifluoromethoxyphenylhydrazone (FCCP), and 0.5 μmol/L Rotenone/antimycin A (Rot/A). There were no significant differences in Basal OCR. (B) Maximal OCR was significantly increased with formoterol‐treated control (CTRL) myoblasts. Maximal OCR was significantly lower in CBA/SIV/ART+ (‐FORM) compared to CTRL. CBA/SIV/ART+ (+FORM) was not different from CTRL. *P < 0.05 from CTRL (‐FORM). (C) There was a significant increase in the expression of PGC‐1a at 6 h after formoterol treatment in CTRL myoblasts. (D) There was a significant increase in mitochondrial DNA copy number (mtDNA/Nuclear DNA expression) at 24 h after formoterol treatment in CTRL myoblasts. There was a 1.6‐fold increase (*P* = 0.07) in formoterol treated CBA/SIV/ART+ myoblasts. *P < 0.05. Data are expressed as mean ± SEM. *N* = 3–4/group.

Because maximal OCR of CBA/SIV/ART+ myoblasts was significantly lower than control myoblasts, the effects of formoterol, a long‐acting β2 agonist, on parameters of mitochondrial bioenergetics and biogenesis were determined. Myoblasts from control and CBA/SIV/ART+ macaques were cultured for 24 h in the presence of 30 nmol/L formoterol for mitochondrial bioenergetics measures using the Seahorse analyzer. There were no differences in basal OCR, proton leak, or spare respiratory capacity among any of the treatment groups (Fig. 3A). As shown earlier, maximal OCR was significantly reduced in CBA/SIV/ART+ myoblasts compared to control cells, and formoterol treatment restored the maximal OCR to 75% of control myoblasts (Fig. 3A and B). In vitro formoterol treatment for 6 h resulted in a twofold increase in PGC‐1α mRNA expression in control myoblasts. However, PGC‐1α expression was not significantly changed in formoterol‐treated CBA/SIV/ART+ myoblasts (Fig. 3C). In vitro formoterol treatment for 24 h significantly increased mtDNA copy number in control myoblasts. There was a 1.6‐fold increase in mtDNA copy number in CBA/SIV/ART+ myoblasts treated with formoterol compared to vehicle‐treatment (Fig. 3D) but this increase failed to reach statistical significance (*P* = 0.07).

## Discussion

In this study, we examined the effects of chronic binge alcohol (CBA) and antiretroviral therapy (ART) on mitochondrial function in skeletal muscle (SKM) during the asymptomatic phase of simian immunodeficiency virus (SIV) infection. CBA, SIV infection, and ART were associated with decreased SDH activity in type 1 and type 2b fibers, indicating a decrease in oxidative capacity of the muscle. Additionally, myoblasts isolated from CBA/SIV/ART+ macaques had decreased maximal oxygen consumption rate (OCR), while myoblasts isolated from SUC/SIV/ART+ macaques did not show a significant difference from control myoblasts, suggesting that CBA and ART might have an additive adverse effect on mitochondrial respiration. Formoterol treatment of naïve control myoblasts increased PGC‐1α expression, mtDNA quantity, and maximal OCR. Moreover, our results show that formoterol treatment of myoblasts isolated from CBA/SIV/ART+ macaques was partially effective in improving maximal OCR to values not significantly different from those in controls. Taken together, these results provide proof‐of‐principle and supporting evidence that myoblasts are responsive to pharmacological exercise mimetics.

Previously we demonstrated that CBA administration to SIV‐infected macaques results in significantly accentuated skeletal muscle wasting at end‐stage SIV infection in non‐ART treated macaques (Molina et al. 2008), which was characterized by increased skeletal muscle proteasomal activity, attenuation of anabolic pathways (LeCapitaine et al. 2011), a profibrotic milieu (Dodd et al. 2014) dysregulation of epigenomic mechanisms (Simon et al. 2015), and dysregulation and genes associated with mitochondrial function and mitophagy (Duplanty et al. 2017). Subsequently we reported that CBA decreases whole body insulin sensitivity (Ford et al. 2016) and decreased myoblast differentiation potential (Simon et al. 2017b) in asymptomatic SIV‐infected macaques irrespective of ART therapy. We propose that altered mitochondrial homeostasis is likely to contribute to the accentuated myopathy observed in CBA‐administered and SIV‐infected macaques, because normal mitochondrial function is critical for normal myogenesis (Wagatsuma and Sakuma 2013) and for maintaining the metabolic homeostasis (Kastaniotis et al. 2017; Karakelides and Nair 2005).

Alcohol, HIV and ART independently have been shown to produce mitochondrial dysfunction, but until now no studies had elucidated their interactions. This is particularly relevant since the incidence of AUD is higher in PLWH than in the general population. Excessive alcohol consumption induces damage to mitochondrial DNA and increases cellular oxidative stress, an important mechanism in the pathogenesis of alcoholic muscle myopathy (Hoek et al. 2002; Bonet‐Ponce et al. 2015). Alcohol metabolism leads to mitochondrial genome degradation in the brain, heart, and skeletal muscle (Mansouri et al. 2001) and decreases markers of mitochondrial biogenesis (TFAM and NRF‐1) (Smiles et al. 2016). Moreover, HIV infection induces mitochondrial damage (Ferri et al. 2000) and long‐term ART has been linked to mitochondrial toxicity and impairment of genes responsible for mitochondrial biogenesis (Falco et al. 2002; Selvaraj et al. 2014). Nucleoside reverse transcriptase inhibitors (NRTIs) have been shown to decrease both mitochondrial DNA and RNA (MTCO3) content in myoblasts and myotubes (Saitoh et al. 2008). Azidothymidine (AZT)‐induced myopathy is associated with increased SDH activity, possibly reflecting mitochondrial accumulation (Simon et al. 2015, 2017b). We propose that these alterations in mitochondrial homeostasis are associated with impaired whole body metabolism and studies supporting this hypothesis show that in HIV+ insulin‐resistant children the basal respiration, ATP production, proton leak, maximal respiration, and spare respiration capacity are lower than those measured in HIV+ noninsulin‐resistant controls (Takemoto et al. 2017).

Reports in the literature on the impact of chronic alcohol consumption on mitochondrial homeostasis are inconsistent. Alcoholics with neurological manifestations of AUD demonstrated muscle mitochondrial respiratory dysfunction (Haida et al. 1998). In contrast, long‐term alcohol consumption did not appear to affect muscle oxidative phosphorylation, respiratory chain complex activity, or cytochrome content, indicating that alcoholic myopathy was not associated with a deficiency in mitochondrial energy supply (Cardellach et al. 1992). Preclinical studies show that rats fed a chronic alcohol diet did not show defects in the mitochondrial energy supply (Cardellach et al. 1991). However, mean mitochondrial respiratory rates were significantly lower in chronic alcohol‐treated animals and in patients with chronic alcoholic myopathy, and this was associated with glycolytic deficiency and a superimposed mitochondrial failure, resulting in a severe energy crisis (Trounce et al. 1990). Moreover, aged HIV+ men on ART treatment, have reduced oxidative enzyme activity (hydroxy acyl‐CoA dehydrogenase (HAD) and citrate synthase (CS)) without significant changes in SDH activity (Ortmeyer et al. 2016). Thus the mitochondrial phenotype resulting from chronic alcohol consumption, HIV infection, and ART remains to be fully elucidated. The results from our controlled studies provide additional insight into the alterations associated with these three conditions.

Formoterol, a long‐acting β2‐adrenergic receptor agonist has previously been shown to stimulate expression of PGC‐1α and increase mitochondrial respiration and biogenesis (Wills et al. 2012). In mice, formoterol increased SKM expression of PGC‐1α and mtDNA content, and significantly improved mitochondrial skeletal muscle oxidative processes and mitochondrial respiration (Sullo et al. 2013). Exercise‐induced increases in PGC‐1α expression were shown to be significantly prevented with β2‐adrenergic receptor antagonist pretreatment, suggesting that the beneficial effects of exercise on mitochondrial homeostasis are partially mediated through ß‐adrenergic receptor stimulation (Miura et al. 2007). Studies show that in vitro formoterol treatment is capable of increasing maximal OCR and mitochondrial copy number (Sullo et al. 2013), leading us to hypothesize that formoterol would improve mitochondrial respiration in myoblasts isolated from CBA/SIV/ART+ macaques. Our results support a beneficial effect of β2 adrenergic stimulation on mitochondrial biogenesis and maximal OCR. Clinical studies show that PLWH undergoing an exercise regimen, had significant increases in VO_2_ max and peripheral blood mononuclear cell mitochondrial respiratory capacity, spare respiratory capacity, and nonmitochondrial respiration (Kocher et al. 2017). We predict that similar improvements may be attained in PLWH with AUD, and this is the focus of current studies from our group.

A limitation to the study is the small sample size. However, it is to be recognized that these studies are conducted in higher primates (closest to human setting) that in contrast to inbred rodents, share similar biological variability with humans. Therefore, when differences are observed that reach statistical significance, despite the small number of animals, we believe these have biological relevance. Moreover, the controlled conditions under which the NHP experiments are conducted enhance the ability of obtaining outcome measures that truly reflect the manipulated variables. Also, to improve reproducibility, all the myoblast studies described have been conducted at least three times and in triplicate for each primary line.

Collectively, our results show that chronic alcohol administration impairs mitochondrial function in myoblasts isolated from SIV‐infected macaques as shown by the decreased skeletal muscle oxidative capacity and maximal myoblast respiration capacity. The isolated myoblasts were not cultured in the presence of alcohol indicating a certain memory of the in vivo myoblast environment (i.e., alcohol, SIV, and ART exposure) that remains after isolation. The effectiveness of formoterol in increasing mitochondrial biogenesis and partially restoring the maximal respiratory capacity of CBA/SIV/ART+ myoblasts together with published clinical work on exercise and the role of adrenergic agonist is evidence that physical activity can improve SKM mitochondrial function. Future studies will test the prediction that aerobic exercise can improve mitochondrial function, oxidative capacity and thus functional skeletal muscle mass in PLWH with AUD.

## Conflict of Interest

None declared.
